# Serratia marcescens endophthalmitis after pterygium surgery: a case report

**DOI:** 10.1186/s12886-017-0590-4

**Published:** 2017-11-02

**Authors:** Myeong Yeon Yi, Jin Kwon Chung, Kyung Seek Choi

**Affiliations:** Department of Ophthalmology, Soonchunhyang University Seoul Hospital, Soonchunhyang University College of Medicine, 59 Daesagwan-ro, Yongsan-gu, Seoul, 140-743 Korea

**Keywords:** Serratia marcescens, Endophthalmitis, Pterygium surgery

## Abstract

**Background:**

To report a case of Serratia marcescens endophthalmitis after pterygium surgery using the bare sclera technique with mitomycin C (MMC).

**Case presentation:**

A 69-year-old male patient underwent pterygium excision surgery using the bare sclera technique and 0.02% MMC. The patient presented with decreased visual acuity and pain from the day after the operation. Trans pars plana vitrectomy was performed and intravitreal antibiotics were administered. Cultures from the aqueous humor and intraocular lens were all positive for S. marcescens, which was sensitive to an empiric antibiotic regimen. The best corrected distant visual acuity, 1 month after treatment, was a finger count/50 cm, but the retinal layer structure and the vasculature were relatively well preserved.

**Conclusions:**

This is the first reported case of S. marcescens endophthalmitis after pterygium surgery. Endophthalmitis caused by S. marcescens has a devastating visual prognosis and may show a high clinical risk-benefit ratio for the application of MMC in pterygium surgery.

## Background

Pterygium surgery is considered to be a safe procedure despite its high recurrence rates. The intraoperative and postoperative use of mitomycin C (MMC) as a surgical adjuvant to prevent recurrence after pterygium surgery has increased. However, potential complications of pterygium surgery with adjuvant MMC, including glaucoma, scleral calcification, infectious scleritis, scleral melting, scleral perforation, iritis, and endophthalmitis have been reported [1–9]. The bare sclera technique is a surgical procedure that excises the head and body of the pterygium while allowing the exposed bare scleral bed to re-epithelialize. This method is similar to regional cosmetic conjunctivectomy to achieve a whitened appearance by leaving the sclera bare and producing a large avascular zone. This procedure was practiced between 2009 and 2011, but is now considered as an inappropriate treatment because of the high rates of significant complications [10]. Postoperative endophthalmitis is a rare but devastating complication after pterygium excision surgery. To the best of our knowledge, this represents the first reported case of endophthalmitis as a complication of the bare sclera technique with MMC therapy.

## Case presentation

A 69-year-old male patient was referred to our hospital for pain and decreased visual acuity in his left eye that started 10 days prior to his visit. Ten days prior to his referral, the patient had pterygium excision surgery in both eyes at an outside clinic. As the medical information from that clinic, complete resection of the pterygium was performed, leaving bare sclera exposed and suturing the conjunctival edge to the underlying sclera using #7-0 vicryl. Sponges soaked with 0.02% MMC were applied intraoperatively over the bare sclera for 2 min. His medical history was significant for well-controlled hypertension, a history of renal transplantation, and taking of the immunosuppressant drug, cyclosporin. There was no ocular history except cataract surgery about 10 years ago.

**Fig. 1:**
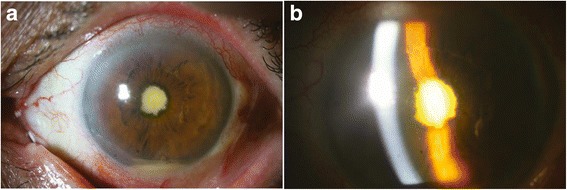
Slit-lamp photographs of anterior segment on initial examination. **a** 1.0 mm hypopyon and 360° posterior synechiae of the iris. **b** Deep central AC with severe corneal edemas and AC inflammation

**Fig. 2:**
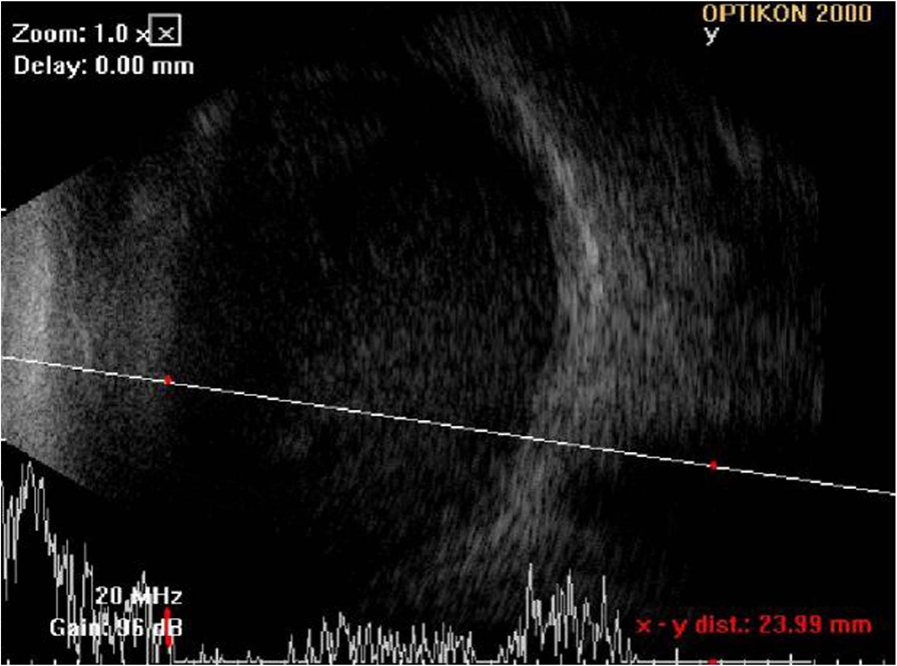
B-scan ultrasonography demonstrated dense vitreous opacities, and a mildly thickened choroid

On initial examination, distant visual acuity with no light perception had an intraocular pressure of 10 mmHg in the left eye. Slit-lamp examination showed diffuse conjunctival injection, nonepithelialized thinned bare sclera over the pterygium excision site and diffuse corneal edema in the left eye, but there was no significant infiltration at the cornea or sclera. A deep anterior chamber (AC) with a 1.0 mm hypopyon and 360° posterior synechiae of the iris were observed (Fig. 1a, b). Fundus examination was impossible because of severe corneal edemas and AC inflammation. B-scan ultrasonography revealed dense vitreous opacities, and a mildly thickened choroid, consistent with acute infectious endophthalmitis (Fig. 2). The right eye was normal.

The patient was admitted to the hospital, where he was treated with systemic (vancomycin 1.0 g every 12 h and ceftazidime 1.0 g every 8 h) and topical (fortified vancomycin 5% and ceftazidime 5%) antibiotics every hour while awake. In spite of extensive investigations including blood culture, ultrasonography of the abdomen, and echocardiography – to exclude the possibility of endogenous endophthalmitis – there was no obvious source of infection. He underwent a pars plana vitrectomy, pars plana intraocular lens removal, membrane peel, air-fluid exchange, intravitreal vancomycin (1.0 mg/0.1 mL) and ceftazidime (2.25 mg/0.1 mL), and silicone oil tamponade the following day. A sample of aqueous humor and intraocular lens was sent for gram stain and culture. During surgery, almost the entire retina was found to be necrotic and covered with a whitish exudative membrane. KOH stain smear revealed no fungus. After 5 days of incubation, the cultures were positive for Serratia marcescens. The organism was more sensitive to ceftazidime than moxifloxacin or gentamycin. Therefore, the patient was continued on systemic ceftazidime (1.0 g every 8 h), topical moxifloxacin 0.5% every hour and prednisolone 1% (every 6 h).

**Fig. 3:**
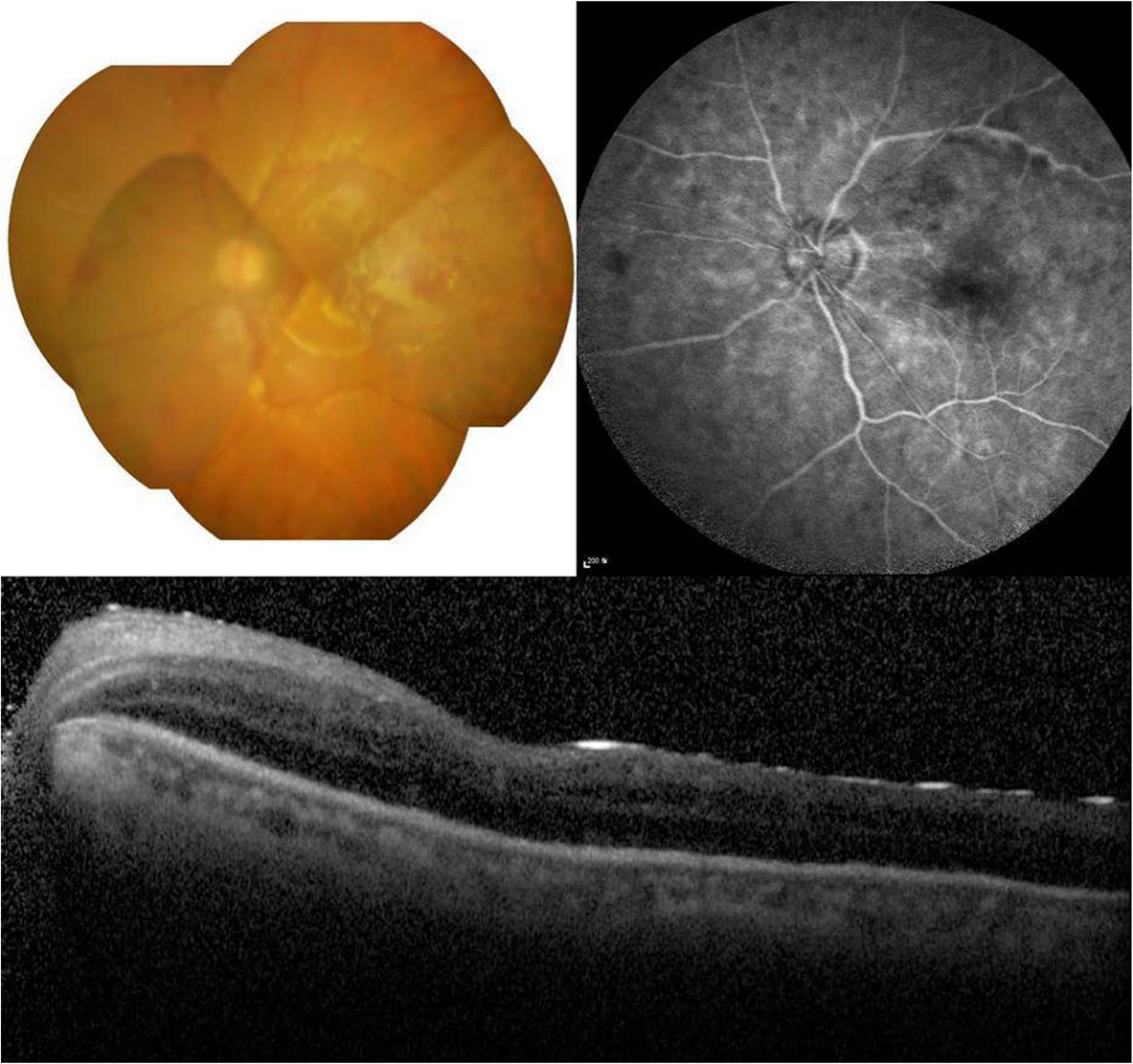
Fundus photograph, fluorescein angiography (FA), and optical coherence tomography (OCT) in the left eye at 1 month after surgery revealed flattened retina with relatively well-preserved retinal layer structure and vasculature

After 1 month, the vision remained at finger count/50 cm but the retinal layer structure and vasculature were relatively well-preserved (Fig. 3).

## Discussion

This report is the first of early-onset S. marcescens endophthalmitis after pterygium surgery with MMC. S. marcescens is a gram-negative bacterium of the family found in water, soil, and the gastrointestinal and urinary tracts of humans; S. marcescens ocular infections are common in immunocompromised hosts [11]. Exogenous S. marcescens endophthalmitis has been noted after cataract extraction, glaucoma procedures, penetrating keratoplasty, and scleral buckle procedures [12].

As a type of alkylating agent, MMC inhibits the synthesis of DNA, RNA, and protein and prohibits fibroblast proliferation [13]. It has been reported that intraoperative administration of MMC is associated with delayed (more than 1 week) conjunctival wound healing [13–16]. It has also been proposed that MMC inhibits the proliferation of inflammatory cells, and induces persistent epithelial defects, thereby diminishing the immune response and increasing the risk of infection [17].

A previous report emphasized severe complications of cosmetic wide conjunctivectomy with topical MMC treatment and raised awareness among clinicians about the risks of the surgical procedure [10]. They suggested that the causes of complications were disruption of the normal conjunctival physiology and destruction of feeding vessels for sclera, resulting in avascular sclera. There are some similarities between cosmetic conjunctivectomy and the bare sclera technique, which can cause avascular sclera and delayed healing. Localized ischemia at the surgical site can be caused by an avascular scleral bed of bare sclera [18]. Leaving wider bare sclera provides a better cosmetic appearance, but long-standing exposure of the sclera with insufficient conjunctival coverage makes it to be more susceptible to infection [19].

The pathophysiology of endophthalmitis after pterygium surgery is not well understood, but the presence of a bare sclera and prevention of conjunctival regrowth, possibly caused by the use of antimetabolites, have been suggested to play a role [20]. In re-epithelization of the conjunctiva, type IV collagen of the basement membrane plays a major role. The sclera is mainly composed of type I collagen and such a different type of collagen between conjunctiva and sclera can cause delayed healing of the conjunctiva epithelium [21]. Delayed conjunctival wound closure could lead to a higher incidence of postoperative infectious complications [10, 14, 22].

We related the patient’s complication to nonepithelialized bare sclera of the conjunctival excision wound site, which was caused by delayed conjunctival wound healing. Transient colonization of the ocular surface by S. marcescens may have allowed and accelerated extensive bacterial colonization through the weakened barrier of the sclera. The pterygium excision wound site lacked conjunctival coverage, and may have contributed to the route to the development of endophthalmitis.

With respect to this patient, the potential factors causing the development of endophthalmitis included a history of renal transplantation (immunocompromised host), and a delayed referral to our clinic. Although the correct concentration and duration of MMC were used, this patient developed an endophthalmitis. Therefore, the use of intraoperative or postoperative MMC and the bare sclera technique in pterygium surgery should be fully examined and re-epithelization of the conjunctival wound is the most important factor to lower the possibility of pterygium complications after surgery.

## Conclusions

Endophthalmitis caused by S. marcescens has a devastating visual prognosis and may show a higher clinical risk-benefit ratio for the application of MMC in pterygium surgery. The importance to the bare sclera technique with MMC of exercising extreme caution cannot be emphasized enough in the setting of primary pterygium removal.

## Abbreviations

AC: Anterior chamber
FA: Fluorescein angiography
MMC: Mitomycin-C
OCT: Optical coherence tomography
S. marcescens: Serratia marcescens

## References

[1] Carrasco MA, Rapuano CJ, Cohen EJ, Laibson PR (2002). Scleral ulceration after preoperative injection of mitomycin C in the pterygium head. Arch Ophthalmol.

[2] Dadeya S, Fatima S (2003). Corneoscleral perforation after pterygium excision and intraoperative mitomycin C. Ophthalmic Surg Lasers Imaging Retina.

[3] Dougherty PJ, Hardten DR, Lindstrom RL (1996). Corneoscleral melt after pterygium surgery using a single intraoperative application of mitomycin-C. Cornea.

[4] Gündüz K, Günalp I, Özden RG (1997). Anterior segment ischemia following pterygium surgery. Jpn J Ophthalmol.

[5] Norliza WW, Raihan IS, Azwa JA, Ibrahim M (2006). Scleral melting 16 years after pterygium excision with topical Mitomycin C adjuvant therapy. Cont Lens Anterior Eye.

[6] Peponis V, Rosenberg P, Chalkiadakis SE, Insler M, Amariotakis A (2009). Fungal scleral keratitis and endophthalmitis following pterygium excision. Eur J Ophthalmol.

[7] Rubinfeld RS, Pfister RR, Stein RM, Foster CS, Martin NF, Stoleru S, Talley AR, Speaker MG (1992). Serious complications of topical mitomycin-C after pterygium surgery. Ophthalmology.

[8] Safianik B, Ben-Zion I, Garzozi H (2002). Serious corneoscleral complications after pterygium excision with mitomycin C. Br J Ophthalmol.

[9] Singh G, Wilson MR, Foster CS (1988). Mitomycin eye drops as treatment for pterygium. Ophthalmology.

[10] Rhiu S, Shim J, Kim EK, Chung SK, Lee JS, Lee JB, Seo KY (2012). Complications of cosmetic wide conjunctivectomy combined with postsurgical mitomycin C application. Cornea.

[11] Johnson D, Klein N, Cunha B (1992). Postoperative Serratia Marcescens endophthalmitis. Heart Lung.

[12] Cohen SM, Flynn HW, Miller D (1997). Endophthalmitis caused by Serratia Marcescens. Ophthalmic Surg Lasers Imaging Retina.

[13] Lam DS, Wong AK, Fan DS, Chew S, Kwok PS, Tso MO (1998). Intraoperative mitomycin C to prevent recurrence of pterygium after excision: a 30-month follow-up study11The authors have no proprietary interest in any of the materials used in this study. 22The protocol was approved by the ethics committee of the Chinese University of Hong Kong. Ophthalmology.

[14] Cano-Parra J, Diaz-Llopis M, Maldonado MJ, Vila E, Menezo JL (1995). Prospective trial of intraoperative mitomycin C in the treatment of primary pterygium. Br J Ophthalmol.

[15] Frucht-Pery J, Siganos CS, Ilsar M (1996). Intraoperative application of topical mitomycin C for pterygium surgery. Ophthalmology.

[16] Panda A, Das GK, Tuli SW, Kumar A (1998). Randomized trial of intraoperative mitomycin C in surgery for pterygium. Am J Ophthalmol.

[17] Long T, Li Z (2015). Bare sclera resection followed by mitomycin C and/or autograft limbus conjunctiva in the surgery for pterygium: a meta-analysis. Int J Ophthalmol.

[18] Alsagoff Z, Tan DT, Chee S (2000). Necrotising scleritis after bare sclera excision of pterygium. Br J Ophthalmol.

[19] Tarr K, Constable I (1980). Late complications of pterygium treatment. Br J Ophthalmol.

[20] Lindquist TP, Lee WB (2015). Mitomycin C-associated Scleral Stromalysis after Pterygium surgery. Cornea.

[21] Krachmer J, Mannis M, Holland E (2005). Cornea: fundamentals, diagnosis and management.

[22] Tsai Y-Y, Lin J-M, Shy J-D (2002). Acute scleral thinning after pterygium excision with intraoperative mitomycin C: a case report of scleral dellen after bare sclera technique and review of the literature. Cornea.

